# Respiratory syncytial virus infection reduces lung inflammation and fibrosis in mice exposed to vanadium pentoxide

**DOI:** 10.1186/1465-9921-11-20

**Published:** 2010-02-22

**Authors:** Elizabeth A Turpin, Aurita Antao-Menezes, Mark F Cesta, James B Mangum, Duncan G Wallace, Edilberto Bermudez, James C Bonner

**Affiliations:** 1 The Hamner Institutes for Health Sciences, Research Triangle Park, North Carolina 27709, USA; 2 Laboratory of Cellular and Molecular Pathology, National Institute of Environmental Health Sciences, Research Triangle Park, North Carolina, 27709, USA; 3 Department of Environmental and Molecular Toxicology, North Carolina State University, Raleigh, North Carolina 27695, USA

## Abstract

**Background:**

Vanadium pentoxide (V_2_O_5_) exposure is a cause of occupational bronchitis and airway fibrosis. Respiratory syncytial virus (RSV) is a ubiquitous pathogen that causes airway inflammation. It is unknown whether individuals with pre-existing respiratory viral infection are susceptible to V_2_O_5_-induced bronchitis. We hypothesized that respiratory viral infection will exacerbate vanadium-induced lung fibrosis.

**Methods:**

In this study we investigated the effect of RSV pre- or post-exposure to V_2_O_5 _in male AKR mice. Mice were pre-exposed by intranasal aspiration to RSV or media vehicle prior to intranasal aspiration of V_2_O_5 _or saline vehicle at day 1 or day 7. A parallel group of mice were treated first with V_2_O_5 _or saline vehicle at day 1 and day 7 then post-exposed to RSV or media vehicle at day 8.

**Results:**

V_2_O_5_-induced airway inflammation and fibrosis were decreased by RSV pre- or post-exposure. Real time quantitative RT-PCR showed that V_2_O_5 _significantly increased lung mRNAs encoding pro-fibrogenic growth factors (TGF-β1, CTGF, PDGF-C) and collagen (Col1A2), but also increased mRNAs encoding anti-fibrogenic type I interferons (IFN-α, -β) and IFN-inducible chemokines (CXCL9 and CXCL10). RSV pre- or post-exposure caused a significantly reduced mRNAs of pro-fibrogenic growth factors and collagen, yet reduced RNA levels of anti-fibrogenic interferons and CXC chemokines.

**Conclusions:**

Collectively these data suggest that RSV infection reduces the severity of V_2_O_5_-induced fibrosis by suppressing growth factors and collagen genes. However, RSV suppression of V_2_O_5_-induced IFNs and IFN-inducible chemokines suggests that viral infection also suppresses the innate immune response that normally serves to resolve V_2_O_5_-induced fibrosis.

## Introduction

A variety of metal oxides cause occupational lung diseases referred to as pneumoconioses. Vanadium is a transition metal commonly found in various types of ores, coals, and oil [1]. Vanadium pentoxide (V_2_O_5_), the most common form of vanadium, is the primary form found in industrial exposure situations [2]. In addition, atmospheric emissions released from power plants that burn coal and oil contribute ~64,000 metric tons of vanadium into the atmosphere each year [2]. Occupational exposure to V_2_O_5 _dust is common in coal-burning power plants and individuals exposed to inhaled V_2_O_5_-containing fly ash suffer from chronic bronchitis and reduced lung function [3,4]. The consequences of environmental exposure to lower levels of V_2_O_5 _on human health remain unclear, in part because air pollution particulates are a complex mixture of many organic and inorganic components, including a variety of metals [5]. However, epidemiologic evidence indicates that individuals at greatest risk for exposure to particulate air pollution are those with pre-existing respiratory diseases such as asthma and viral bronchitis [6,7].

Respiratory syncytial virus (RSV) is a ubiquitous virus that causes airway inflammation and bronchitis [8,9]. The virus is an enveloped negative-sense single-stranded RNA Paramyxovirus of the subfamily *Pneumonidae *[10]. Since its isolation, RSV has been identified as a leading cause of epidemic respiratory tract illness in children in the United States and worldwide [11]. Although RSV exposure in the human population occurs at a very early age, immunity is incomplete after RSV infection and secondary infections can occur throughout life. Airway epithelial cells are the primary target of RSV infection, and they respond to the infection by producing a variety of mediators involved in lung immune/inflammatory responses, such as cytokines, chemokines, and interferons [12].

Occupational bronchitis and airway fibrosis caused by V_2_O_5 _is recapitulated in rats or mice exposed by intratracheal instillation or pharyngeal aspiration [13,14]. In these studies, V_2_O_5 _causes airway and interstitial fibrosis that partially resolves within several weeks after exposure. Profibrogenic growth factors, including platelet-derived growth factor (PDGF) and its receptor, are elevated in rats exposed to V_2_O_5 _[15]. The PDGF system plays a pivotal role in orchestrating myofibroblast migration and proliferation at sites of forming fibrotic lesions [16]. Moreover, tyrosine kinase inhibitors selective for PDGF or EGF receptors reduce V_2_O_5_-induced fibrosis in rats [17]. The partial resolution of V_2_O_5_-induced fibrotic lung lesions in rodents is due at least in part to the potent action of V_2_O_5 _as an activator of STAT-1, a transcription factor that mediates fibroblast growth arrest and apoptosis [18]. Moreover, STAT-1-deficient mice are more susceptible to pulmonary fibrosis following lung injury [19].

Studies with human cells also indicate that V_2_O_5 _induces both pro-fibrogenic and anti-fibrogenic factors. For example, gene expression profiling of normal human lung fibroblasts exposed to V_2_O_5 _in culture show increased levels of pro-fibrotic and angiogenic growth factors (PDGF, CTGF, HB-EGF, VEGF) as well as protective IFNs [20]. The production of pro-fibrogenic growth factors and anti-fibrogenic IFNs and chemokines is dependent on the generation of reactive oxygen species [21,22]. In general, the production of both pro-fibrogenic and anti-fibrogenic mediators by human lung cells in response to V_2_O_5 _is consistent with a partially resolving lung fibroproliferative response in mice or rats exposed to V_2_O_5 _by a single intratracheal or pharyngeal aspiration.

The hypothesis of this research is that respiratory viral infection will exacerbate vanadium-induced lung fibrosis. In contrast, we report that RSV pre- or post-exposure reduces V_2_O_5_-inflammation, cell proliferation, and fibrosis in male AKR mice. Moreover, RSV pre- or post-exposure significantly reduced mRNA levels of pro-fibrogenic growth factors and collagen, and yet also reduced RNA levels of anti-fibrogenic interferons and CXC chemokines. Collectively these data suggest that RSV infection reduces the severity of V_2_O_5_-induced fibrosis by suppressing pro-fibrogenic growth factors and collagen genes. However, RSV suppression of V_2_O_5_-induced IFNs and IFN-inducible chemokines also suggests that viral infection has a negative effect on the immune response triggered by V_2_O_5 _exposure. These results have potentially important ramifications, since a wide variety of metal oxides cause occupational lung diseases and RSV infection is commonplace.

## Materials and methods

### Animals

Six-week old male pathogen-free CDF AKR mice were purchased from The Jackson Laboratory (Bar Harbor, ME) and housed in an Association for Assessment and Accreditation of Laboratory Animal Care (AAALAC)-accredited facility that was humidity and temperature controlled. AKR mice were tested as they are susceptible to both RSV and metal induced bronchitis [23]. Mice were housed in microisolator cages on Alpha-dri cellulose bedding and supplied water and cereal-based diet NIH07 (Zeiger Brothers., Gardners, PA) ad libitum. The animal studies were approved by The Hamner Institutes for Health Sciences Institutional Animal Care and Use Committee.

### Experimental design

**Figure 1:**
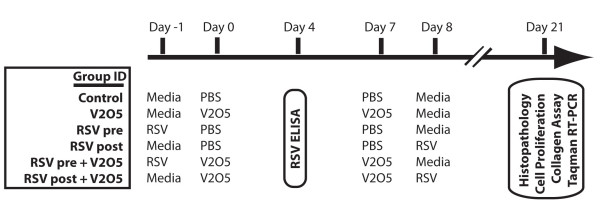
**Experimental Design for exposing adult male AKR mice to RSV and V_2_O_5_**. A pilot group of mice received an intranasal exposure to RSV or media vehicle alone to test for infection in the lungs at day 4. All other histopathology and gene expression endpoints listed were assessed at 21 days following intranasal exposure to RSV (6 × 10^5 ^PFU) and/or V_2_O_5 _(4 mg/kg) as described in Methods.

Animals were randomly assigned to treatment groups (seven mice per group) and acclimated for two weeks. Treatment groups included a media control (media collected from non-inoculated Vero cells), V_2_O_5_, RSV pretreatment, RSV post-treatment, RSV pretreatment+V_2_O_5_, or RSV post-treatment+V_2_O_5 _(Fig. 1). RSV strain A2 (a kind gift from Dr. Ralph Tripp, University of Georgia, Athens) was propagated in Vero cells and concentrated using Amicon Ultra filters (Millipore Corporation, Bedford, MA). RSV was diluted to 6 × 10^5 ^PFU in a 100 μl dose and inoculated intranasally into both nares on days -1 and 8 under light isoflurane anesthesia. A pilot group of mice were exposed to RSV and lung harvested at day 4 to determine the presence of RSV in whole lung tissue by ELISA as described below. V_2_O_5 _(Sigma-Aldrich Chemical, St. Louis, MO) was suspended in PBS and sonicated for 30 minutes. Mice were given intranasal inoculation of 50 μl of V_2_O_5 _(4 mg/kg) or PBS into both nares on days 0 and 7 under light isoflurane anesthesia. Preliminary studies demonstrated that intranasal administration of either RSV or V_2_0_5 _resulted in even distribution in all lung lobes (data not shown). Negative control animals were treated with PBS. Animals were euthanized at 21 days following initial particle exposure.

### Necropsy and preparation of lung tissues

One hour prior to euthanasia, mice received a single intraperitoneal injection of 50 mg/kg body weight of bromodeoxyuridine (BrdU; Sigma-Aldrich). The lungs were lavaged with PBS as described below, the right lung lobes were snap-frozen in liquid nitrogen and stored at -80°C and used for RNA isolation and collagen assay as described below. The left lungs were pressure-infused intratracheally at 20 cm H_2_0 with 10% neutral-buffered formalin. The same lungs were lavaged and sampled for histopathology, collagen, quantitative PCR, and ELISA assays to allow for direct comparison of assay results. Lungs were fixed for approximately 48 hours and then changed into 70% ethanol. Three cross-sectional portions of the left lung were embedded in paraffin, sectioned at 5 μm, and stained with Masson's trichrome, hematoxylin and eosin, or immunostained for BrdU as described below.

### Bronchoalveolar lavage

Mice were euthanized by pentobartital overdose and lungs were lavaged five times with 1-ml volumes of PBS. Bronchoalveolar lavage fluid (BALF) collected from the first two recovered lavages were pooled and placed on ice. The subsequent three lavages were pooled and placed on ice. BAL cells collected by centrifugation were resuspended in culture medium and enumerated using an automated cell counter (Model ZM, Coulter, Marietta, GA). Cytospins were prepared with 10^5 ^cells per slide. Cell differential counts, performed on HEMA-3 (Fisher Scientific, Pittsburgh, PA) stained cytocentrifuge slide preparations, were based on a total number of 350 cells. Total protein and lactate dehydrogenase (LDH) in cell-free BALF from the first two pooled lavages were analyzed spectrophotometrically using a COBAS FARA II (Roche Diagnostic Systems Inc., Montclair, NJ).

### Lung histopathology and pathology scoring

The lungs from the 21 day time point were scored for fibrosis according to our previously reported method [24]. The left lung lobe was formalin-fixed, embedded in paraffin and cut in 5-μm sections. The lungs were scored for the amount of collagen present (based on Masson's trichrome-stained sections), the thickness of the alveolar walls, and the number of fibroblast-like cells associated with the lesions. Sections were scored blindly on a relative scale where zero represented the levels of these parameters in the PBS control group, 1 representing minimal fibrosis, 2 representing mild fibrosis, 3 representing moderate fibrosis, 4 representing marked fibrosis, and 5 representing severe fibrosis.

### Bromodeoxyuridine (BrdU) immunohistochemistry and Cell Proliferation

Cell labeling indices were determined in the bronchiolar/alveolar region and in the bronchus-associated lymphoid tissue for each animal, and the mean labeling index was calculated for each group of eight animals. The BALT was examined as it is believed to facilitate primary immune response to respiratory infection and would serve as a marker for inflammation in the lung [25].

### RSV ELISA

Lung samples from mice were analyzed on an antigen capture ELISA modified from previously described protocols [26,27]. A goat anti-RSV polycolonal antibody (0601, ViroStat, Portland, ME) was diluted 1:500 in Tris-buffered saline (TBS), 100 μl added per well and incubated overnight at 4°C. Plates were washed with TBS+ 0.05% Tween 20 (TBST) and blocked with 200 μl of TBST with 1% BSA for 1 h at room temperature. Plates were washed three times to remove blocking buffer. Lung homogenates were prepared by homogenizing the right accessory lung lobe in 500 ml of PBS for 5 s with a tissue homogenizer. Lung homogenates were diluted 1:5 and 100 μl added per well in duplicate and incubated overnight at 4°C. After washing, 100 μl of a biotin conjugated anti-RSV polyclonal antibody (0607, ViroStat) diluted 1:500 in TBST with 1% BSA was added and incubated for 1 hr at room temperature. Plates were then washed three times and 100 μl of ExtrAvidin-peroxidase (E2886, Sigma-Aldrich) diluted 1:1000 in TBST with 1% BSA. Plates were washed three times and detected with 3,3',5,5'-Tetramethylbenzidine (TMB, T5524, Sigma-Aldrich). After 30 min incubation, the reaction was stopped with 0.5 N H_2_SO_4_. The values are expressed as the OD observed at 450 nm.

### Collagen Assay

The right cranial lobe of each mouse lung was suspended in PBS at 50-100 mg tissue per ml and homogenized for 10 s with a Tissuemiser homogenizer (Fisher Scientific). Cellular debris was pelleted by centrifugation and the supernatant analyzed for total protein with the BCA Assay Kit (Pierce/ThermoFisher Scientific, Rockford, IL) according to the manufacturer's instructions. The Sircol™ Soluble Collagen Assay kit (Biocolor Ltd., Northern Ireland) was used to extract collagen from duplicate samples by using 100 μl of supernatant mixed with acetic acid and 500 μl of Sircol Dye Reagent according to the manufacturer's instructions. Similarly prepared collagen standards (10-50 μg) were run in parallel. Collagen was pelleted by centrifugation; pellets were washed twice with denatured alcohol and dried prior to suspension in Alkali reagent. Absorbance at 540 nm was read on a Multiskan EX microplate spectrophotometer (ThermoFisher Scientific) microplate reader with Ascent software. Data were expressed as μg of soluble collagen per mg of total protein.

### Taqman quantitative RT-PCR

Total RNA from the right anterior lung was isolated using TRIZOL reagent (Invitrogen, Carlsbad, CA), followed by RNA cleanup performed using RNeasy Midi spin columns (Qiagen, Valencia, CA). One microgram of total RNA was reverse transcribed at 48°C for 30 minutes using Moloney murine leukemia virus reverse transcriptase (Eurogentec, San Diego, CA) in 1× RT buffer, 5 mM MgCl_2_, 500 μM of each dNTP, 2.5 μM of random nonamers, and 0.4 U/μL RNase inhibitor in a volume of 100 μl. Twenty nanograms of the RT product was amplified using Taqman Gene Expression Assays specific for platelet-derived growth factor receptor alpha (PDGFRα), PDGF-A, PDGF-C, transforming growth factor beta-1 (TGF-β1), connective tissue growth factor (CTGF), type I procollagen (COL1A2), vascular endothelial growth factor (VEGF) and 18S on the Applied Biosystems 7900 Prism^® ^Sequence Detection System (Applied Biosytems, Foster City, CA). The PCR conditions and data analysis were performed according to the manufacturer's protocol described in User bulletin no.2, Applied Biosystems Prism 7700 Sequence Detection System. Gene expression was measured by the quantitation of cDNA converted from mRNA corresponding to the target genes relative to the vehicle-treated control groups and normalized to eukaryotic 18S reference endogenous control. Relative quantitation values (2^-ΔΔCT^) were expressed as fold-change over controls.

### Data and statistical analysis

All graphs were constructed and statistical analysis performed using GraphPad Prism^® ^software v. 5.00 (GraphPad Software, Inc., San Diego, CA). A one-way ANOVA with a post-hoc Tukey test was used to identify significant differences among treatment groups.

## Results

**Figure 2:**
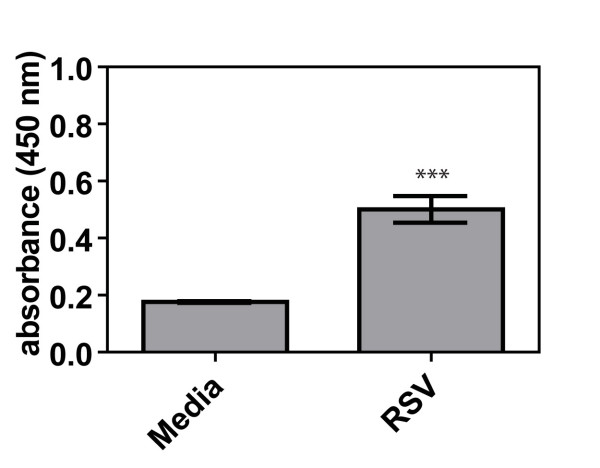
**Detection of RSV in the lungs of male AKR mice 4 days after a single intranasal aspiration of virus-containing media or media vehicle alone**. The presence of RSV in the lungs was measured by ELISA as described in Methods. Data were analyzed by Student's t-test. ***P < 0.001 compared to Media.

We first sought to determine whether RSV would infect the lungs of AKR mice after a single intranasal inoculation in the absence of any V_2_O_5 _exposure. Using an RSV-specific ELISA, we found that the intranasal delivery of RSV caused infection in the lungs of mice after 4 days (Fig. 2). An optical density (O.D.) value of 0.2 represented no virus. RSV infection was accompanied by a transient inflammatory response in the airways of AKR mice observed at 4 days (data not shown). Collectively these data show that the intranasal inoculation of RSV resulted in viable, replicating RSV within the lungs of AKR mice. Lung samples were also analyzed at day 21 in all mice and no RSV was detected (data not shown) as would be expected. In addition, the effect of V_2_O_5 _on RSV replication was evaluated *in vitro *using cultured human lung epithelial H292 cells. In these *in vitro *experiments, V_2_O_5 _treatment had no effects on virus replication with pre or post treatment (data not shown).

**Figure 3:**
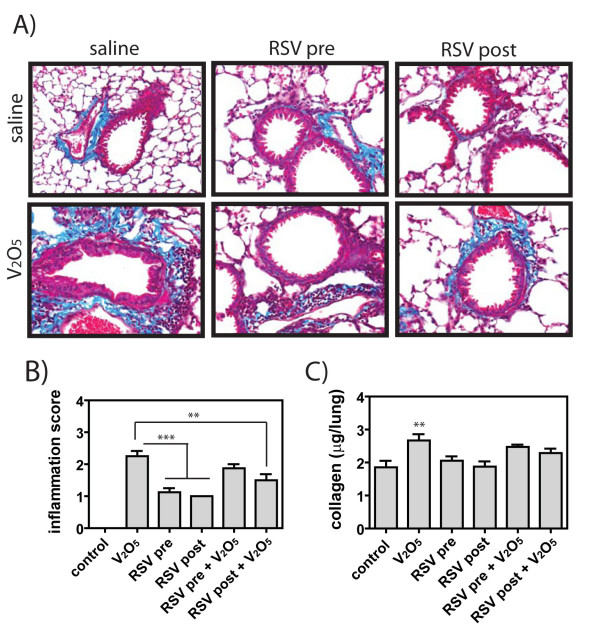
**Lung histopathology at 21 days in male AKR mice treated with RSV and/or V_2_O_5_**. (**A**) Representative airway pathology in mice exposed to V_2_O_5 _with or without RSV pre- or post-exposure. Lung sections were stained with Masson's trichrome to show collagen (blue staining). (**B**) Inflammation scores were determined in a blinded manner as described in Methods. Data were analyzed by One-way ANOVA followed by Tukey's test. **P < 0.01 or ***P < 0.001 compared to V_2_O_5_. (**C**) Lung collagen was measured by Sircol™ assay as described in Methods. Data were analyzed by One-way ANOVA followed by Tukey's test. **P < 0.01 compared to Control.

**Figure 4:**
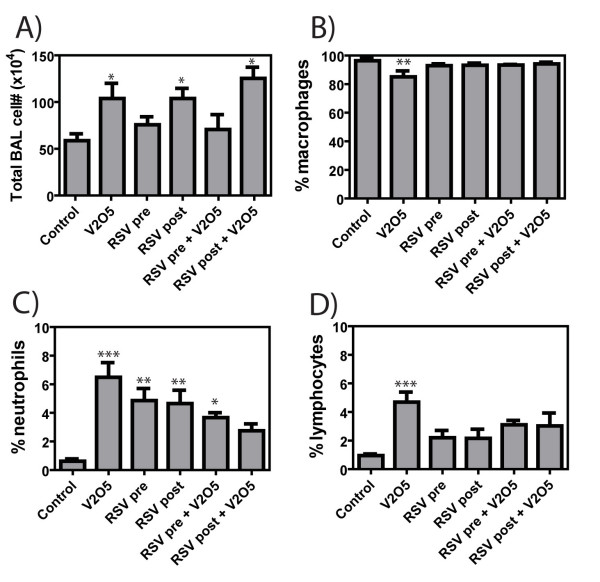
**Cell differential counts in bronchoavleolar lavage (BAL) fluid at 21 days after exposure to V_2_O_5 _in the absence or presence of RSV pre- or post-exposure showing total numbers of BAL cells recovered by lavage (A) the relative percentages of (B) macrophages, (C) neutrophils, and (D) lymphocytes**. Data were analyzed by One-way ANOVA followed by Tukey's multiple comparison test. *P < 0.05, **P < 0.01, or ***P < 0.001 compared to Control.

Airway fibrosis, increased airway wall thickness due to increased collagen and/or fibroblasts around the airways, as well as interstitial fibrotic lesions within the lung parenchyma, increased septal thickening due to collagen and/or fibroblasts in the alveolar region, was significantly increased by 21 days following a single intranasal exposure to V_2_O_5 _(Fig. 3A). The airway fibrotic response to V_2_O_5 _exposure in AKR mice was qualitatively less severe in mice that received either pre- or post-RSV exposure, whereas RSV exposure alone did not appear different from media control exposed control lung tissue at 21 days (Fig. 3A). In a blinded pathology evaluation of the lung sections, the V_2_O_5_-induced inflammation score was significantly reduced by RSV post-exposure, whereas RSV pre-exposure had no effect on the V_2_O_5_-induced inflammation score (Fig. 3B). The lungs of the V_2_O_5 _treated mice had increased inflammation composed of increased total numbers of cells as well as increased numbers of neutrophils and lymphocytes. In addition, V_2_O_5 _significantly increased total lung collagen levels as measured by Sircol assay, but not when mice were pre- or post-exposed to RSV (Fig. 3C). V_2_O_5 _treatment increased the total BAL numbers when give alone or with RSV post-exposure, while RSV pre-exposure and RSV pre-exposure+V_2_O_5 _levels were similar to controls (Fig 4). V_2_O_5 _increased the numbers of neutrophils and lymphocytes in BAL fluid at 21 days post-exposure mirroring what was observed in lesion scoring of lungs (Fig. 4). RSV exposure alone also increased the numbers of BAL neutrophils and lymphocytes. However, RSV pre- or post-exposure reduced the V_2_O_5_-induced increase in these inflammatory cells by ~50% (Fig. 4).

**Figure 5:**
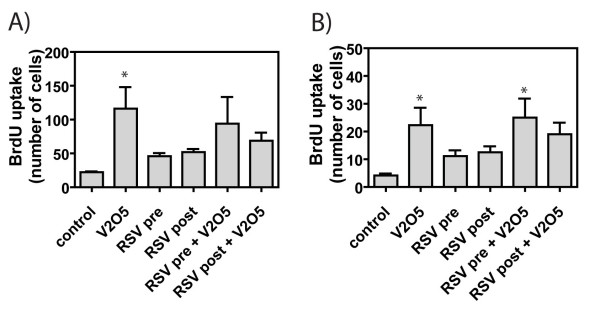
**Cell proliferation in the lungs of male AKR mice at 21 days after exposure to V_2_O_5 _in the absence or presence of RSV pre- or post-exposure**. Bromodeoxyuridine (BrdU)-positive cells were quantified in (**A**) lung parenchyma and (**B**) airways as described in Methods. Data were analyzed by One-way ANOVA followed by Tukey's multiple comparison test. *P < 0.05 compared to Control.

Cell proliferation, as assessed by BrdU-positive nuclei, was significantly increased around airways and in the lung parenchyma of mice 21 days after V_2_O_5 _exposure (Fig. 5). BrdU-positive epithelial and sub-epithelial mesenchymal cells were observed in the airways and lung parenchyma of mice exposed to RSV and/or V_2_O_5 _(data not shown). BrdU labeling was > 1% in the lungs of control animals. RSV exposure alone also caused an increase in cell proliferation, although the number of BrdU-positive cells were one-third to half of that observed for V_2_O_5 _alone. RSV pre- or post-exposure did not significantly change the cell proliferation index in either airways or lung parenchyma (Fig. 5).

**Figure 6:**
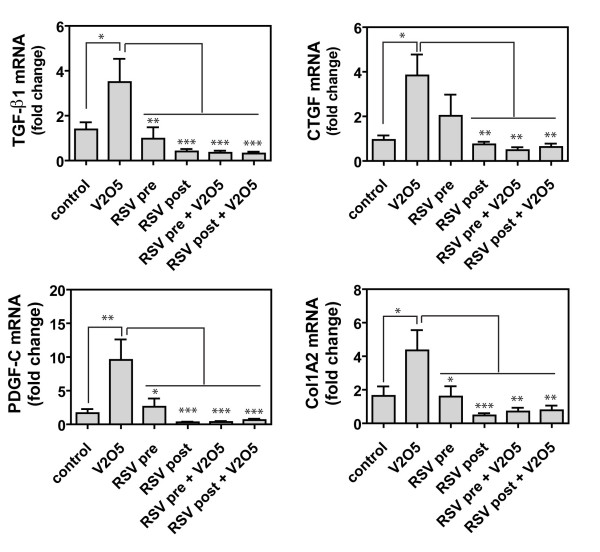
**Quantification of mRNAs encoding growth factors (TGF-β1, CTGF, PDGF-C) and collagen (Col1A2)**. The mRNA levels for each growth factor or collagen was measured by Taqman quantitative real-time RT-PCR. Data were analyzed by One-way ANOVA followed by Tukey's test. Comparisons were V_2_O_5 _versus Control or V_2_O_5 _versus other treatments as indicated. *P < 0.05, **P < 0.01, ***P < 0.001.

We next sought to determine the effect of RSV on the mRNA levels of growth factors and collagen induced by V_2_O_5_. The mRNAs encoding several pro-fibrogenic growth factors (TGF-β1, CTGF, and PDGF-C) were significantly increased by V_2_O_5 _exposure in whole lung tissue at 21 days post-exposure (Fig. 6). RSV exposure alone did not significantly increase the mRNA levels of any of these three growth factors nor was the collagen (Col1A2) mRNA level increased by RSV alone. However, RSV pre- or post-exposure completely inhibited V_2_O_5_-induced growth factor and collagen mRNA levels at 21 days (Fig. 6).

**Figure 7:**
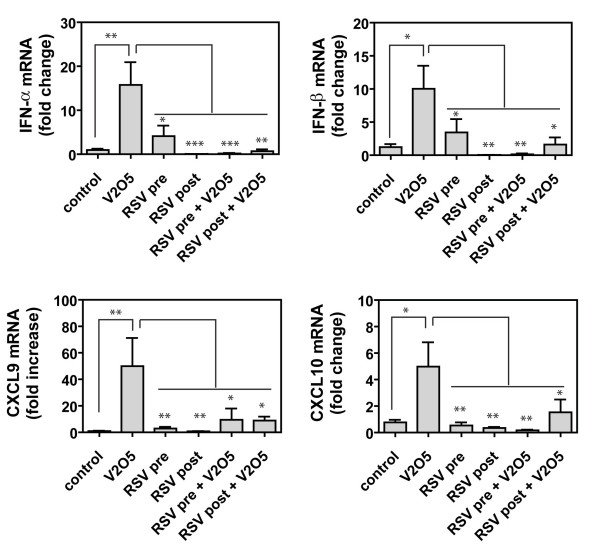
**Quantification of mRNAs encoding type I interferons (IFN-α, IFN-β) and CXC chemokines (CXCL9 and CXCL10)**. The mRNA levels for each cytokine or chemokine was measured by Taqman quantitative real-time RT-PCR. Data were analyzed by One-way ANOVA followed by Tukey's test. Comparisons were V_2_O_5 _versus Control or V_2_O_5 _versus other treatments as indicated. *P < 0.05, **P < 0.01, ***P < 0.001.

Since IFNs and IFN-inducible CXC chemokines have been suggested to play a role in the resolution of V_2_O_5_-induced fibrogenesis, we also assessed the effect of RSV on mRNA levels encoding type I IFNs (IFN-α and IFN-β) and downstream chemokines CXCL9 (Mig) and CXCL10 (IP10). The mRNAs encoding (IFN-α, IFN-β, CXCL9, and CXCL10 were significantly increased by V_2_O_5 _exposure in whole lung tissue at 21 days post-exposure (Fig. 7). RSV exposure alone marginally increased mRNA levels of IFN-α and IFN-β, but did not increase CXCL9 or CXCL10 mRNAs at 21 days. RSV pre- or post-exposure completely blocked V_2_O_5_-induced mRNA levels of IFN-α, IFN-β, CXCL9, and CXCL10 (Fig. 7).

## Discussion

RSV is known to exacerbate airway diseases such as cystic fibrosis and asthma [28-30]. Moreover, the toxicity of air pollutant particles is generally thought to be increased by viral infections, and conversely, some air pollutant particles increase viral infections [31,32]. Nevertheless, the effect of respiratory viral infection on the pathogenesis of occupational lung diseases caused by the inhalation of metals has not been previously investigated to our knowledge. We hypothesized that RSV infection would increase airway fibrosis and remodeling caused by V_2_O_5_, a transition metal that causes occupational bronchitis in individuals working in coal and oil-burning power plants. Surprisingly, we found that RSV infection before V_2_O_5 _exposure reduced the lung inflammation score, whereas RSV infection after V_2_O_5 _significantly reduced lung inflammation as compared to metal exposure alone. RSV pre- or post-exposure significantly reduced V_2_O_5_-induced increases in neutrophil and lymphocyte infiltration into the lung. Furthermore, V_2_O_5 _exposure alone significantly increased lung collagen, whereas we observed no significant increase in lung collagen in mice that received RSV before or after V_2_O_5_.

The reduction in V_2_O_5_-induced fibrosis by RSV infection was accompanied by a marked reduction in mRNAs encoding pro-fibrogenic growth factors and collagen. Exposure to V_2_O_5 _in the absence of RSV increased TGF-β1 and collagen mRNA levels several-fold after 21 days of exposure. TGF-β1 has long been regarded as the most potent stimulator of collagen synthesis during lung fibrosis [33]. RSV exposure alone caused no induction of TGF-β1 or collagen mRNAs at 21 days and completely suppressed V_2_O_5 _induction of TGF-β1 and collagen mRNAs. CTGF and PDGF-C, which also mediate collagen deposition and/or fibroblast survival [16,34], were induced by V_2_O_5 _several-fold and induction was blocked by RSV at 21 days. Therefore, the overall pattern of RSV effect on V_2_O_5_-stimulated responses was to block pro-fibrogenic signaling. A limitation of our analysis was that we assayed mRNA levels at 21 days post-V_2_O_5 _exposure. It is possible that RSV caused changes in mRNA levels encoding these pro-fibrogenic mediators at earlier time points and returned to baseline levels of expression by 21 days.

The effect of RSV pre-exposure or post-exposure was previously studied in combination with carbon black (CB) ultrafine particles using BALB/C mice [26,35]. In experiments where mice were first exposed to CB and then RSV, viral titers in RSV + CB mice were lower than RSV alone on days 2-4 of infection, and yet by day 7 of expsoure neutrophil numbers, proinflammatory cytokine mRNA expression, and protein levels of TNF-alpha and the Th2 cytokine interleukin (IL)-13 were increased in the lungs of RSV + CB mice, indicating an exacerbation of infection [26]. These data indicated that pre-exposure to ultrafine particles induces an allergic (Th2) immune response rather than an IFN-γ-mediated (Th1) response production necessary for microbial defense. In a second study by the same investigators, mice were intratracheally instilled with CB particles after 3 days of RSV infection [35]. Neutrophil and lymphocyte numbers were higher on days 4 and 14 of infection in CB-exposed, RSV-infected mice. CB exposure also enhanced RSV-induced airway hyperresponsiveness to methacholine, bronchoalveolar lavage (BAL) total protein, and virus-associated chemokines monocyte chemoattractant protein (MCP-1), macrophage inflammatory protein (MIP-1 alpha), and regulated upon activation, normal T cell expressed and secreted (RANTES). These data demonstrated a synergistic effect of ultrafine CB particles on RSV infection, and suggest a potential mechanism for increased respiratory infections in human populations after PM exposure. In contrast to the studies by Lambert and coworkers described above, our data show that either pre- or post-exposure to RSV reduced V_2_O_5_-induced lymphocyte and neutrophil influx into the lung, decreased pro-fibrogenic growth factor and collagen mRNAs, and reduced inflammation and fibrosis.

As mentioned above, the toxic effects of air pollution particles is generally thought to be increased by viral infections [31]. For example, diesel exhaust particles enhance influenza virus infection in human airway epithelial cells and the enhanced susceptibility to diesel exhaust by influenza infection is associated with decreased surfactant protein expression [32,36]. However, the interactive effects between virus and pollutant particle could largely depend on the type of virus and the composition of the pollutant particle in question. Air pollution particles are a complex mixture of organic (e.g., carbon, polycyclic aromatic hydrocarbons) and inorganic constituents (e.g., transition metals) [5]. Vanadium-containing air pollution particles are released at the highest levels in oil-burning power plants that release residual oil fly ash into the atmosphere [6]. These vanadium-containing fly ash particles then contribute to urban ambient particulates (UAP). Our data in mice exposed to pure V_2_O_5 _suggest that pre-exposure to RSV infection would not increase the risk to vanadium-containing UAP. However, it would be important to determine whether RSV would exacerbate the effects of vanadium-containing UAP in the lung or whether other types of viral infections (e.g., influenza) would increase lung inflammation or fibrosis caused by vanadium-containing UAP or pure V_2_O_5_.

Viral infection has been implicated in the pathogenesis of idiopathic pulmonary fibrosis (IPF). While little is known about the mechanisms of IPF, the disease is likely multi-factorial, and viruses have been implicated as co-factors (either as initiating or exacerbating agents) of fibrotic lung disease [37]. Specifically, experimental data suggests a link between hepatitis C virus, adenovirus, human cytomegalovirus and the Epstein-Barr gammaherpesvirus, in IPF [38]. Therefore, despite the findings of our study, viral infections should be considered as potential initiators or exacerbating agents in at least some cases of IPF and possibly other types of occupational and environmental lung diseases that involve fibrotic responses.

The mechanism(s) through which V2O5 exerts a fibrogenic effect in the lungs of mice is likely complex and further confounded by RSV exposure. While V_2_O_5 _clearly has pro-fibrogenic activity in the lungs of mice and increased growth factor levels that mediate fibrosis, V_2_O_5 _also increased type I IFNs (IFN-α and IFN-β) along with IFN-inducible chemokines CXCL10 and CXCL9. Furthermore, we observed that RSV blocked V_2_O_5_-induced increases in IFN and CXC chemokine mRNAs. IFNs could be protective against fibrosis by causing growth arrest and apoptosis of fibroblasts, and CXCL10 has been reported to have angiostatic properties [39]. We have also reported that V_2_O_5 _is a potent activator of type I IFN production by lung fibroblasts that leads to STAT-1 activation and proposed this mechanism in the resolution V_2_O_5_-induced fibrosis [22]. The RSV surface attachment protein (G protein) has been shown to inhibit Toll-like receptor (TLR) 3/4-mediated IFN-β induction [40] a feature that may facilitate virus replication. In the present study, we found that RSV reduces V_2_O_5_-induced mRNA levels of type I IFNs, CXCL9 and CXCL10. Therefore, while RSV reduces V_2_O_5_-induced fibrosis in AKR mice, this effect is limited by the action of RSV in also reducing potentially protective IFNs and chemokines.

Finally, it may be noteworthy that certain metals, including vanadium, possess broad spectrum anti-viral activity that includes inhibition of RSV replication [41]. We did not specifically address the issue of viral replication in the lungs of mice exposed to V_2_O_5 _in the present study. An important focus of future study will be to determine if RSV or other viruses (e.g., influenza) are inhibited by V_2_O_5 _or vanadium-containing air pollutant particles.

## Conclusions

The intranasal inoculation of mice with RSV clearly did not exacerbate V_2_O_5_-induced pulmonary inflammation and fibrosis, but rather reduced these pathologic endpoints and reduced V_2_O_5_-induced neutrophil and lymphocyte infiltration into the lungs. Moreover, RSV pre- or post-exposure significantly reduced mRNA levels of pro-fibrogenic growth factors and collagen, and yet also reduced RNA levels of anti-fibrogenic interferons and CXC chemokines. Collectively these data suggest that RSV infection reduces the severity of V_2_O_5_-induced fibrosis by suppressing pro-fibrogenic growth factors and collagen genes. However, RSV suppression of V_2_O_5_-induced IFNs and IFN-inducible chemokines also suggests that viral infection has a negative effect on the immune response triggered by V_2_O_5 _exposure. Our findings in mice suggest that RSV infection does not increase the risk of occupational bronchitis caused by vanadium exposure. Further studies should address whether occupational lung disease caused by the inhalation of metals other than vanadium are modulated in a positive or negative way by RSV infection.

## List of Abbreviations

RSV: respiratory syncytial virus; V_2_O_5_: vanadium pentoxide; PDGF: platelet-derived growth factor; TGF: transforming growth factor; CTGF: connective tissue growth factor; IFN: interferon; CXCL: CXC-motif ligand chemokine; STAT-1: signal transducer and activator of transcription.

## Competing interests

The authors declare that they have no competing interests.

## Authors' contributions

EAT and JCB designed the experiments, performed the data analysis, and drafted the manuscript. EAT, AAM, JBM, DGW, and EB performed mouse necropsy, BAL, RNA isolation and Taqman quantitative real-time RT-PCR. MFC performed histopathology analysis and scoring. All authors read and approved the final manuscript.
